# Evaluation of Volatile Profile, Fatty Acids Composition and in vitro Bioactivity of *Tagetes minuta* Growing Wild in Northern Iran

**DOI:** 10.15171/apb.2018.014

**Published:** 2018-03-18

**Authors:** Farshid Rezaei, Rashid Jamei, Reza Heidari

**Affiliations:** Department of Biology, Faculty of Science, University of Urmia, West Azerbaijan, Iran.

**Keywords:** Tagetes minuta, GC-MS analysis, Antioxidant capacity, Fatty acid, HPLC-UV

## Abstract

***Purpose:*** The aim of the present study was to investigate the chemical properties of wild Tagetes minuta L. (family Astreacea) collected from Northern Iran during the flowering period concerning the chemical combination of the essential oil along with its antioxidant properties and composition of fatty acids.

***Methods:*** The essential oil of the plant was extracted by a Clevenger approach and analyzed using gas chromatography-mass spectroscopy (Capillary HP-5ms GC/MS Column). Fatty acid contents of this species as a result of hexane extraction were analyzed by means of gas chromatography (GC-FID) while their phenolic contents were analyzed by high performance liquid chromatography (HPLC-UV). In this research also the total polyphenolic (TPC) and total flavonoid (TFC) content was determined spectrophotometrically while the antioxidant activity was evaluated using the DPPH (2,2'-diphenyl-1-picrylhydrazyl) bleaching method.

***Results:*** GC/MS analysis of the essential oil identified monoterpenoid fractions (52.13%) as the main components and among them dihydrotagetone (23.44%) and spathulenol (10.56%) were the predominant compounds. The evaluation of fatty acid content revealed that saturated acids were prevailing compounds and the major components are: palmitic (30.74±0.4%) and capric (24.15±0.5%) acids. Chromatographic separation of its phenolic contents indicated that this herb contain sinapic acid derivatives rather than hydroxybenzoic acid derivatives. Also the essential oil showed an effective antioxidant capacity (TPC=153.27±0.9 mg/g, TFC=63.79±0.1 mg/g, IC_50_ = 29.31±0.8 µg/ml).

***Conclusion:*** The results proved that the plant could be used for nutritional and pharmaceutical purposes.

## Introduction

*Tagetes minuta* from sunflower (Asteraceae - Helenieae) family is a species native to South America but is now a widespread weed in most parts of the world.^1^* T. minuta* is reported to contain a number of bioactive metabolites of high medicinal, industrial and nutritional value.^2,3^ The majority of published studies of *T. minuta* focused on the chemodiversity of volatile oils, flavonoids and thiophenes.^4^ Etheric oils are a source of bioactive and valuable molecules that are used in many fields such as aromatherapy, cosmetic, pharmaceutical, nutrition, agronomic and perfume industries.^5^ The essential oil compositions of* T. minuta* has been studied previously in Iran and other parts of the world.^6-11^ From these findings, it could be seen that the variation in Tagetes oil depends on several environmental as well as genetic factors.^12,13^ In addition, according to different activities of various bioactive compounds, these differences between components of the essential oil will be important in pharmaceutical and nutritional uses.^14^ All these scientific facts, make a thorough evaluation necessary of the native “Tagetes oil” at regional level. Scientific evidence suggests that vegetable oils as another important class of phytochemicals play a vital role in a healthful diet.^15^They provide energy and essential fatty acids (linoleic and linolenic acids), which are necessary for good health. The oils are also crucial to the absorption of the fat-soluble vitamins A, D, E, and K.^15^ The amount of unsaturated fats is one of the most important parameter of different edible vegetable oils.^16^ On the other hand, the influence of common environment and genes on the fatty acid composition of plants has been demonstrated by several researchers.^17,18^ Previously published investigations on *Tagetes* species have demonstrated strong antioxidant properties.^19,20^ Phenol and related compounds occur in food products, especially those of plant origin and are known to be responsible for the antioxidant activities. These secondary metabolites are the subject of increasing scientific interest because of their importance in human health.^21^ In recent years, a number of researchers have reported that environmental factors (soil type, sun exposure and rainfall) can widely affect phenolic compounds of the foods.^21^ However, since the biosynthesis of volatile compounds are affected by geographical variation and there is no report on the composition of the essential oil of *T.minuta* from North of Iran, also no study has been carried out previously on phenolic and fatty acid compounds of this plant grown in Iran, therefore this work was carried out; to identification and compare volatile constituents by GC-MS; to analyze the phenolic acids by HPLC and finally; to investigate quantification of fatty acids in the oil extracted of *Tagetes minuta* by GC which may be used as initial materials for medical purposes and use in relevant industries.

## Material and Methods

### Plant material

*Tagetes minuta* L. plant was collected in early-autumn of 2015 from Roodbar (latitude: 37^º^44^׳^ 20^״^ N ; longitude: 40^º^96^׳^ 44 ^״^E and 180 m above sea level) in Province of Guilan (Noth of Iran). In this time the plant is in full swing, which can be used for the extraction of essential oil. Plant identification was carried out by botanist, Guilan Agricultural Research Center (GARC), Dr. Morady and a voucher specimen of the plant has been deposited (no. 5543). The material was aired for a few days in a well-ventilated and protected from direct sunlight.

### Essential oil isolation

Air-dried aerial parts (flowering stage - 50g) were subjected to hydrodistillation for 3 h using a Clevenger-type apparatus (British type) to extract essential oils. The oil samples obtained were dehydrated over anhydrous sodium sulfate and kept in a cool and dark place until further analyses.

### GC-MS analysis 

The analysis of volatile organic compounds isolated from *T.minuta* was performed using a complete HP/Agilent 6890/5973 GC-MS system with a FID detector (Palo Alto, USA). The GC column specified for the methods was a capillary HP-5MS column (5% phenylmethylsiloxane, 30 m X 0.25 mm i.d., coating thickness 0.25 μm). The injector temperature was set at 250 °C and helium carrier gas flow was adjusted to 1 ml/min. The samples pass through the interface heated to 200 °C into the mass spectrometer operated at 70 eV, which is scanned from 30-300 amu. The electron multiplier voltage was increased to near 3000 V, and the injection volume was 1 µl. GC oven temperature ranged from 70º to 240 °C at l0 °C /min. Qualitative identification for separated constituents was achieved using MS data libraries (Wiley7n.1 and NIST 2008), by comparison of their retention time (RT) obtained on HP-5MS column and then verified by (RI, HP-5MS) with those published in the literature.^22^

### Crude oil extraction and GC analysis

Fatty acid methyl esters (FAMEs) were prepared using 2 mol/l NaOH in methanol and n-heptane. The oil samples were analyzed for chemical components using GC equipped with Flame Ionization Detector (GC-FID, model 6890 N, Agilent Technologies, USA) and DB-Wax capillary column having 30 meter length (30 m, 0.25 mm i.d., 0.25 µm film thickness). It was also fitted to a injector with Agilent tapered liner (4mm id). A fixed quantity of oil (1 μl) was injected into the column after dilution. Oven temperature ranged between 100-230 °C. The temperature of the FID injection system was 250 °C and that of FID detector was 280 °C. Nitrogen carrier gas flow was adjusted to 1.8 ml/min. The identification of individual component was made by running standards (Sigma, Chemical Co.St. Louis). The area under individual peak being calibrated into percent area by integrator itself, gives the percentage of individual component in the sample.

### HPLC-UV separations

Methanol extract (80%) and essential oil of the plant were evaluated in this study. The main Polyphenol standards were used for separation and characterisation of phenolic compounds in the aromatic herb. The analysis of phenolic acids was performed with a HPLC system Knauer-Germany equipped with UV-Vis detector according to the method proposed the authors.^23^ The filtered samples were injected on Eurospher 100–5 C_18_ column (25 cm X 4.6 mm; 5 μm) from Agilent Technologies. In this experiment, the mobile phase for the HPLC-UV system was composed of deionized water/acetic acid (2% v/v) as solvent A and acetonitrile as solvent B at flow rate 0.8 ml/min. The gradient used in the evaluation was as follows: from 0 to 5 min isocratic 85% A flow, from 5 to 19 min a linear gradient of 85% A to 100% B. In this process, fifteen minutes of equilibration (85% A) is required before another injection. The column temperature was maintained at 25°C and the injection volume was 20 μl. Peaks were detected at 280 nm. Finally, phenolic compounds were determined according to their UV spectra, relative retention times and comparison with reference curves.

### Determination of total phenolic content (TPC)

Total phenolic concentration (TPC) in the bioactive extracts namely the methanol extracts and essential oils were estimated spectrophotometrically (WPA Biowave S2100) by the Folin–Ciocalteu (FC) assay described perviously with slight modification.^24^ In this study the TPC was expressed as milligrams of gallic acid equivalents (GAE) per gram dry weight of samples. Briefly, an aliquot of (20 µl) the samples were transferred into a test tube and 1 ml reactive FC (previously diluted 10-fold) was added and mixed. Afterward, 0.8 ml of 7.5% sodium carbonate solution was added to the mixture and mixed gently. After incubation for 30 min in the dark and at ambient temperature (25 °C), the absorbance was read at 765 nm of wave length against blank by using a UV–Visible spectrophotometer. TPC values were calculated from a standard curve plotted with known concentration of gallic acid and all determinations were done in triplicate. (The calibration equation for Gallic acid: y =0.0421 x - 0.0232, R^2^ =0.998).

### Determination of total flavonoid contents (TFC)

Quantification of flavonoids was based on a procedure described by Shin et al. consisting of a spectrophotometric test at 510 nm.^25^ Methanolic solution of extracts and EOs (20 µl) were mixed with 1 ml of distilled water and 0.075 ml of a 5% Sodium nitrite solution. After 5 min, 0.15 ml of 10% aluminium chloride solution was added. It was left at room temperature for 6 min and 0.5 ml of sodium hydroxide (1 mol/l) was added. Then the absorbance of each mixture was measured immediately at 510 nm with a double beam UV/Visible spectrophotometer. The calibration curve was plotted by preparing quercetin solutions in methanol. (The calibration equation for quercetin: y = 0.0779 x - 0.0136, R^2^ = 0.9979).

### DPPH (2,2'-diphenyl-1 -picrylhydrazyl) method

The free-radical scavenging activity was measured by the decrease in absorbance of methanolic solution of DPPH.^26^ Briefly, 10 µl of the samples were added to 2 ml of methanolic DPPH (0.0023 mol/l) solution, and allowed to react at room temperature. After 30 min the absorbance values were measured at 520 nm and converted into the percentage antioxidant activity using the following formula:

% inhibition of DPPH radical = 100 – 100 (AS ÷ A0)

where A0 is absorbance of the blank and AS is absorbance of the sample at 520 nm. Also the IC_50_ of the plant was obtained by plotting the percent DPPH remaining at the steady state (60 min) of the reaction against the corresponding antioxidant level. The IC_50_ is the concentration of antioxidant to quench 50% DPPH under the experimental conditions.

### Statistical analysis

Statistical analysis was carried out using SPSS software version 17 (one-way ANOVA and *p* < 0.05). All the samples were done in triplicate, except those for GC-MS method which were analyzed once; The results are expressed as mean values and standard deviation (SD).

## Results and Discussion

### Chemical composition of the essential oil

The results of GC-MS analyses of Tagetes oil is reported in Table 1 for the first time from Northern Iran. The volatile oil (orange-yellow color) obtained by hydrodistillation method of the aerial parts of *Tagetes minuta* showed an average yield of 0.77% (according to dry material weight). The different chemical constituents of the plant were identified and listed in order of their increasing retention times on the HP-5MS column. A total of twenty- four chemical compounds were separated and detected, representing 92.32% of the total oil. Most of the volatile compounds analyzed during this work contain mainly 9 monoterpenes (52.13%) and 15 sesquiterpenes (40.19%).The oil profile exhibits dihydrotagetone (23.44%) as the most abundant compound. The other main compounds were spathulenol (10.56%), caryophyllene oxide (6.35%), alpha-atlantone (4.76%) and isoaromadendrene epoxide (3.11%), respectively. In fact, the essential oil tested was rich in monoterpenoids. The oil of *T. minuta* L. has been evaluated in some parts of the country by a number of investigators who have recognized α-terpineol (20.8%), β-ocimene (17.6%)^6^ and dihydrotageton (45.9%), trans-ocimenone (27.0%), cis-*β*-ocimene (11.9%)^8^ and limonene (13.0%), piperitenone (12.2%), α-terpinolene (11.0%)^7^ as the major components in this species. A review and analysis of the literature revealed that no oil of any *Tagetes minuta* has been found in which spathulenol and caryophyllene oxide were the main ingredients so its identification of potential significance in Iran. Also according to the Articles, no isoaromadendrene epoxide and neophytadiene were reported of the oil. On the other hand, the main chemical substance essential oil obtained from Tagetes oil in England was dihydrotagetone (54.1%).^10^ As high amount of dihydrotagetone found in the oil our plant (23.44%). The main constituents identified in another studies in South Africa and Saudi Arabia were cis-*β*-ocimene and 5 octyn-4-one 2,7-dimethyl (50.9%, 11.52% respectively).^9,10^ On comparison to this reports, the findings of our study indicated the diversity of volatile terpenes and their percentage which can be attributed to ecological, climatic and genetically factors. Here it is worth noting that despite this variations, the main constituents of *T. minuta* oil growing wild in north of Iran are oxygenated monoterpenoids (41.75%) that can be a valuable for further research especially medicinal properties.

### Analysis of fatty acids

In this study, the fatty acid composition of the *T.minuta* were obtained by hexane extraction and analyzed using a GC–FID for the first time from Iran. The fatty acid profiles of the vegetable oil tested are presented in Table 2. A total of 12 fatty acids were detected and quantified. The amount of fatty acid contents of the extracted oil decreased in the order of: saturated fatty acids (SFA) > polyunsaturated fatty acids (PUFA) > monounsaturated fatty acids (MUFA) ranging from 89.35%, 7.63% and 3.02%, respectively. Palmitic acid (16:0) was the most abundant fatty acid in all the samples. In addition to palmitic acid (30.74 ± 0.4%), other abundant fatty acids extracted from the vegetable oil were capric acid (24.15±0.5%), luric acid (16.58±0.7%), myristic acid (11.02±0.4%) and linoleic acid (5.75±0.5%) respectively. The remaining fatty acids were minor in concentrations (Table 2). Of course, in the evaluation linoleic acid (5.75%) and linolenic acid (1.86%) showed considerable amounts. However, comparing between the fatty acid profiles obtained from current study with fatty acids previous reported on this plant some differences were observed.^17^ For example, Ahmad et al. reported that the major constituents of the seed oil were linoleic (51.95%), palmitic (21.64%) and oleic (16.23%) acids.^17^ The differences seem to be associated with genetic factors as well as environmental conditions. However, these findings proved that the plant sources of beneficial fatty acids such as palmitic, louric and linoleic acids. Scientific research has shown that linolenic acid is related to a lower risk of heart patients.^27^

**Table 1:** Chemical composition of the volatile oil of *T.minuta*

**Compounds**	RI*	**Percent**
Sabinen	972	0.8
Limonene	1032	2.38
β-Ocimene	1050	7.2
Dihydrotagetone	1052	23.44
trans-Tagetone	1139	5.76
Camphor	1145	2.05
Z-Tagetone	1148	3.5
Z-ocimenone	1229	4.2
Bornyl acetate	1287	2.8
β-Caryophyllene	1418	1.17
α-Humulene	1455	1.06
Germacrene D	1483	1.29
Caryophyllene oxide	1582	6.35
(*E*)-Nerolidol	1564	0.94
Viridiflorol	1593	0.9
Spathulenol	1576	10.56
Carotol	1596	0.7
Isoaromadendrene epoxide	1602	3.11
E-sesqui-lavandulol	1610	1.76
β-Atlantone	1668	1.42
1682	0.63
α-Atlantone	1786	4.76
Neophytadiene	1840	2.94
Perhydrofarnesyl acetone	1847	2.6
Monoterpene hydrocarbons		41.75
Oxygenated monoterpenes		10.38
Sesquiterpene hydrocarbons		6.46
Oxygenated sesquiterpenes		33.73
**Total**		**92.32**

RI* = Retention indices as determined on HP-5MS column

### HPLC-UV quantitative analysis

We analyzed for the first time the polyphenols from *T.minuta* in Iran. Table 3 shows the standard chromatogram values of 9 individual phenolic substances in the aerial parts of the plant, have been carried out by HPLC-UV. It is noteworthy that the separation of all examined compounds was carried out in 50 min. Nine phenolic compounds were analyzed and eight compounds were determined: 4-hydroxy benzoic acid, caffeic acid, syringic, vanillic acid, *p*-coumaric acid, ferulic acid, caffeic acid and rutin (flavonoid glycosides). By examining the HPLC profiles we observe that sinapic acid (5.86±0.1 mg/g) and 4-hydroxy benzoic acid (0.79±0.07 mg/g) are predominant in volatile oil and methanolic extract, respectively. Also gallic acid is not in the samples. However, the pattern of polyphenols indicated that the plant contains sinapic acid derivatives rather than hydroxybenzoic acid derivatives. Our results was supported by several studies in the literature reported that leaves of *T.minuta* exhibits high levels of hydroxycinnamic acid and quercetin derivatives (32±2 and 10±1 mg/g dw, respectively).^28^ In fact, phenolic acids have gained considerable attention because of their potential protective role against oxidative damage diseases.^21^

**Table 2:** Amount of various fatty acids in *T.minuta* oil

**No**	**Fatty acid**	**Acronym**	**Concentration (%)**
1	Capric acid	C10:0	24.15 ± 0.5
2	Lauric acid	C12:0	16.58 ± 0.7
3	Myristic acid	C14:0	11.02 ± 0.4
4	Palmitic acid	C16:0	30.74 ± 0.4
5	Palmitoleic acid	C16:1	1.19 ± 0.3
6	Stearic acid	C18:0	0.24 ± 0.05
7	Oleic acid	C18:1	0.01 ± 0.03
8	**Linoleic acid**	C18:2	5.75 ± 0.5
9	**Linolenic acid**	C18:3	1.86 ± 0.1
10	Arachidic acid	C20:0	5.3 ± 0.5
11	Behenic acid	C22:0	1.32 ± 0.2
12	Erucic acid	C22:1	1.82 ± 0.2
	TSFA (Total saturated fatty acids)		89.35
	TUFA (Total un saturated fatty acids)		10.65
	TUFA/TSFA		0.1

Each value is the mean ± SD of three independent measurements

### Determination of total polyphenols and flavonoid contents

The Folin–Ciocalteu reagent is widely used for the colorimetric in vitro assay of polyphenolic antioxidants. Plant phenolics constitute a large group of secondary metabolites which act as primary antioxidants.^29^ As shown in Table 4, total phenolic contents in the oil of *T.minuta* was 153.27±0.9 mg GAE*/*g dry plant sample. Lower total phenolic contents were found in the methanol extract of the plant (34.17±0.6 mg GAE/g dry extract). In the past years a study performed by Ranilla et al. on selected leaves showed 67±7 0.6 mg GAE/g of phenolic compounds in the dry weight of the extracts (methanolic) of *Tagetes minuta*.^28^ Our results seem to be consistent with other research which found *Tagetes minuta* is rich of phenolic acids and can be considered as a invaluable source of natural antioxidants.

In terms of TFC, the oil of *T. minuta* (63.79±0.1 mg/g, *p* < 0.05) was richer in flavonoids, than the extract (14.86±0.4 mg/g, *p* < 0.05) (Table 4). Previously, Kaisoon et al. discovered that the flowers of this genus had 68.9 mg/g of flavonoid content.^30^ Flavonoids are a large family of plant polyphenolics that act as free-radical scavengers and the biological function is related to their chemical structure (hydroxyl groups).^31^

### Antioxidant activity

The DPPH method is an simple, rapid, valid accurate and sensitive approach to evaluate the antioxidant activity of a special composition or plant extracts.^32^Especially, EO of *T.minuta* (DPPH=66.23±0.92%, IC_50_=29.31±0.8 µg/ml) has more antioxidant potential than its methanolic extracts (DPPH=36.21±1.44%, IC_50_=48.67±0.8 µg/ml) (Table 4). This antioxidant activity is in line with those of previous studies with I_C50_ between 35-344 μg/ml.^19^ Thus, the study suggests that these fractions (The methanol extract and the volatile oil) are good sources of antioxidant compounds. However, the extraction techniques and time of extraction can highly affect the results of DPPH assay.^33^

**Table 3:** Content of phenolic compounds in essential oil (**A**) and methanolic extract (**B**) of *T.minuta*

**No**	**Phenolic compound**	Calibration curve*	**R** ^ 2 ^	**Plant material (mg/g DW)**	
				**A**	**B**
1	Rutin	Y=2e+06x-1e+06	0.998	-	0.36 ± 0.02
2	Galic acid	Y=2E+06x-2E+06	0.997	-	-
3	Caffeic acid	Y=1E+06x-2E+06	0.988	2.82 ± 0.06^a^	0.75 ± 0.06^b^
4	4- Hydroxy benzoic acid	Y=838158x-1E+06	0.998	2.96 ± 0.08^a^	0.79 ± 0.07^a^
5	Vanillic acid	Y=3E+06x-29609	0.999	0.27 ± 0.05^a^	0.05 ± 0.01^a^
6	P-coumaric acid	Y=82.887x-59041	0.998	1.58 ± 0.02^a^	0.65 ± 0.1^a^
7	Syringic acid	Y=13571x-3682.9	0.985	4.62 ± 0.1^a^	0.18 ± 0.03^b^
8	Ferulic acid	Y=165138x-136553	0.988	2.63 ± 0.05^a^	0.74 ± 0.09^b^
9	Sinapic acid	Y=20727x-9590	0.997	5.86 ± 0.1^a^	0.56 ± 0.1^b^

* Linear calibration curves for the HPLC-UV analysis of the phenolic compounds. Each value is presented as the mean ± SD (n=3). Mean values in the same row followed by a different letter (a,b) are significantly different (*p* <0.05)

**Table 4:** The content of total polyphenols, flavonoids and antioxidant capacity parameters in the plant

**Samples**	**TPC (mg GAE/g)**	**TFC (mg QUE/g)**	**DPPH (%)**	**IC**_50_ **(µg/mL)**
EO	153.27 ± 0.9^a^	63.79 ± 0.1^a^	66.23 ± 0.92^b^	29.31 ± 0.8^b^
Extract	34.17 ± 0.6^b^	14.86 ± 0.4^b^	36.21 ± 1.44^c^	48.67 ± 0.8^a^
Gallic acid	-	-	93.12 ± 0.4^a^	0.15 ± 0.01^c^

Each value is the mean ± SD of three independent measurements. Mean values in the same column followed by a different letter (a-c) are significantly different (*p* <0.05). GAE: Gallic acid equivalents; QUE: Quercetin equivalents

## Conclusion

The present study demonstrates that the volatile oil of *Tagetes minuta* is rich in monoterpenes with two major components dihydrotagetone and spathulenol. The oil tested shows significant quantitative and qualitative variations when compared with previous reports from Iran and abroad. The differences could be possibly due to genetic variation and environmental factors. This research also revealed that the aerial parts of this aromatic plant are various sources of oily components, especially the essential ones, as well as of good natural sources of unsaturated fatty acids (such as linoleic acid). On the other hand, it was found that the native *Tagetes* oil is a non negligible source of phenolic compounds. Therefore, in addition to traditional uses this medicinal Plant can also be grown and utilized in Pharmaceutical and food industries.

## Acknowledgments

The authors would like to express their gratitude to Scientific Research Projects Coordination Unit of Urmia University for financial support (Project Number: 996/2015/D30).

## Ethical Issues

Not applicable.

## Conflict of Interest

The authors declare no conflict of interest.
